# Effect of Calcination Atmosphere on the Performance of Cu/Al_2_O_3_ Catalyst for the Selective Hydrogenation of Furfural to Furfuryl Alcohol

**DOI:** 10.3390/molecules29122753

**Published:** 2024-06-09

**Authors:** Yongzhen Gao, Wenjing Yi, Jingyi Yang, Kai Jiang, Tao Yang, Zhihan Li, Meng Zhang, Zhongyi Liu, Benlai Wu

**Affiliations:** 1 College of Chemistry, Zhengzhou University, Zhengzhou 450001, China; 2 School of Chemical Engineering, Zhengzhou University, Zhengzhou 450001, China; 3 State Key Laboratory of Coking Coal Resources Green Exploitation, Zhengzhou University, Zhengzhou 450001, China

**Keywords:** furfural, selective hydrogenation, furfuryl alcohol, Cu/Al_2_O_3_ catalyst, calcination atmosphere

## Abstract

The selective hydrogenation of the biomass platform molecule furfural (FAL) to produce furfuryl alcohol (FA) is of great significance to alleviate the energy crisis. Cu-based catalysts are the most commonly used catalysts, and their catalytic performance can be optimized by changing the preparation method. This paper emphasized the effect of calcination atmosphere on the performance of a Cu/Al_2_O_3_ catalyst for the selective hydrogenation of FAL. The precursor of the Cu/Al_2_O_3_ catalyst prepared by the ammonia evaporation method was treated with different calcination atmospheres (N_2_ and air). On the basis of the combined results from the characterizations using in situ XRD, TEM, N_2_O titration, H_2_-TPR and XPS, the Cu/Al_2_O_3_ catalyst calcined in the N_2_ atmosphere was more favorable for the dispersion and reduction of Cu species and the reduction process could produce more Cu^+^ and Cu^0^ species, which facilitated the selective hydrogenation of FAL to FA. The experimental results showed that the N_2_ calcination atmosphere improved the FAL conversion and FA selectivity, and the FAL conversion was further increased after reduction. Cu/Al_2_O_3_-N_2_-R exhibited the outstanding performance, with a high yield of 99.9% of FA after 2 h at 120 °C and an H_2_ pressure of 1 MPa. This work provides a simple, efficient and economic method to improve the C=O hydrogenation performance of Cu-based catalysts.

## 1. Introduction

The energy supply crisis and environmental pollution caused by the excessive consumption of the fossil energy have aroused worldwide concerns [1]. In order to achieve the sustainable development, researchers have spared no efforts to explore and develop new renewable energy as the alternative, which has included solar energy, hydroelectric energy, wind energy, biomass energy, geothermal energy, tidal energy and so on [2,3]. Among them, biomass energy is the only carbon-containing energy and is the fourth largest category after the traditional fossil energy of coal, oil and natural gas [4]. It occupies a very important position in the overall energy system. Compared with other energy products, biomass energy has the advantages of abundant reserves, strong renewable ability and wide distribution and is an ideal substitute for fossil energy [2,5].

**Figure 1:**
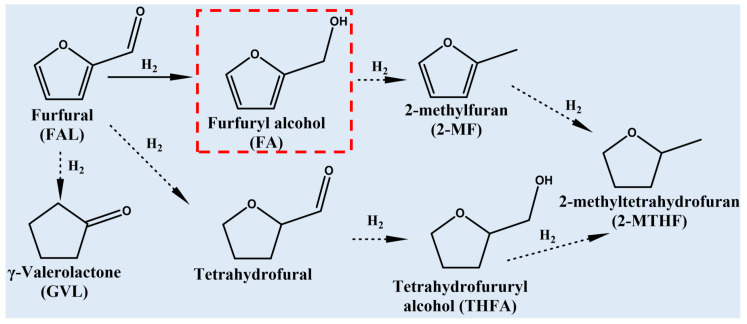
Reaction pathways of FAL hydrogenation to FA and other chemicals.

Furfural (FAL) is an important biomass platform compound and considered to be one of the most promising platform molecules for sustainable production of fuels and chemicals in the 21st century [6,7]. FAL has a variety of functional groups such as furan ring, aldehyde group and diene ether, and it can generate various derivatives through selective hydrogenation reactions [8,9], as shown in Figure 1. Among these products, furfuryl alcohol (FA), as one of the important value-added intermediates, accounts for about 65% of the total output of FAL derivatives [10]. FA is mainly used in the production of resins [8], plasticizers [11], wood preservatives [12] and as a platform molecule for the synthesis of other chemicals [13]. Due to the complexity of the FAL reaction pathway, obtaining high FAL conversion and high FA selectivity simultaneously remains a great challenge [11].

The CuCr oxide catalyst is mainly used in industry to catalyze FAL hydrogenation to produce FA [14]. However, the high conversion and remarkable yield require high temperature and high pressure, and the employed catalysts also produce the toxic Cr-containing waste for the environment [15]. In recent years, developing the Cr-free catalysts with superior performance as the alternatives has been an interesting topic [16]. In the beginning, most of the catalysts used to catalyze the FAL hydrogenation conversion were noble metals, including Pd [17], Pt [18] and Ru [19]. Although the noble metal catalysts have the high intrinsic hydrogenation activity, the low selectivity for FA and high price limit their large-scale industrial application. Recently, non-noble metals, including Fe [20], Co [21], Ni [22] and Cu [23], have attracted wide interest because of their abundant reserves, low price and simple availability. As long as the reactivity, selectivity and stability can be effectively improved, they can be the promising alternatives for the industrial applications [14]. Among the non-noble metals, Cu-based catalysts have received much attention due to their specific hydrogenation activity towards C=O [24]. Theoretical calculations and experimental results have well demonstrated that FAL is linear chemical adsorbed in a η^1^(O) configuration on the active Cu species [14]. This avoids the hydrogenation of the furan ring, resulting in high FA selectivity. However, the low reactivity of Cu-based catalysts urgently needs to be improved [25].

Many strategies, including but not limited to improving the active components [26,27], modulating the supports [28,29], doping the promotors [30,31] and tuning the preparation methods [32,33], have been used to develop the novel Cu-based catalysts. Zhang et al. [26] obtained the highly distributed Cu/MgO catalysts, where the Cu^0^/Cu^+^ molar ratio increased gradually with the upward reduction temperature. The experimental results showed that the Cu/MgO-350 exhibited excellent stability and reusability without significant activity loss. The reduction temperature could modulate the nature of the interface between the Cu^0^/Cu^+^ active species and the metal oxide interface in Cu/MgO catalysts. Gong et al. [34] reported a copper-based catalyst supported by sulfonate group (-SO_3_H)-grafted active carbon (AC). The modified Cu/AC-SO_3_H catalyst exhibited an enhanced catalytic performance for selective hydrogenation of FAL to FA in the liquid phase. Through grafting the sulfonate group on the support, better metal dispersion, more Cu^0^/Cu^+^ species and stronger FAL adsorption capacity were attained to contribute the high hydrogenation performance. Zhang et al. [30] synthesized a Pd-CuO_x_ nanocomposite catalyst with outstanding performance for the selective hydrogenation of FAL to FA. The valence state of Cu and Pd-Cu interactions played the critical roles in determining the intrinsic activity of the prepared Pd-Cu catalysts. Various characterizations combined with the kinetic experiments and in situ chemisorption clearly unraveled the adsorption and activation processes of the C=O bond and H_2_ molecule on Pd^0^, Cu^0^ and Cu^+^ sites. Zhang et al. [35] provided an efficient strategy of K_2_CO_3_ assisting the CuO#TiO_2_ catalyst for the liquid-phase hydrogenation of FAL. The reason for the increased yield was ascribed to the boosted generation of the surface Cu^+^/Cu^0^ active sites and the promoted gaseous hydrogen dissolution in ethanol media by K_2_CO_3_. Cheng et al. [36] developed a new catalytic transfer hydrogenation (CTH) strategy for the continuous conversion of FAL into FA with Cu/ZnO/Al_2_O_3_ as the catalyst under mild conditions. The highly dispersive Cu species (Cu^0^, Cu^+^), with few Lewis acidic sites and η^1^(O)-type adsorption, ensured high selectivity and mild conversion. Du et al. [32] investigated the effect of preparation methods on the structure and performance of Cu/SiO_2_ catalysts. The results showed that the excellent performance was associated with the highest Cu^0^ surface area, the smallest Cu particle size and the suitable Cu^+^/(Cu^+^ + Cu^0^) ratio. The above studies have confirmed that the efficient Cu-based catalysts are closely related to the abundance of Cu^+^ and Cu^0^ species. Cu^0^ and Cu^+^ active species synergistically catalyze C=O bond hydrogenation, where Cu^0^ promotes the dissociation of H_2_, while Cu^+^ adsorbs and activates C=O bonds as the Lewis acid sites [37].

In addition to the above methods, the calcination atmosphere also has great influences on the size of the active metal [38], the metal–support interaction [39] and the surface active species [40], which could also affect the catalytic performance [41]. However, the effect of the calcination atmosphere on the activity of Cu-based catalysts is rarely reported. Moreover, compared with other modification methods, tuning the calcination atmosphere to pretreat catalyst precursor is easier to operate and more economic, which has the potential application prospects.

Herein, Cu/Al_2_O_3_ catalyst precursor prepared by the ammonia evaporation method was calcined in the different atmospheres and employed for FAL hydrogenation. The physical and chemical properties of Cu/Al_2_O_3_ samples were systematically characterized by in situ XRD, TEM, N_2_O titration, H_2_-TPR, and XPS, and the correlation between catalytic activity and catalyst properties was discussed to unravel the effects of calcination atmosphere on the hydrogenation performance.

## 2. Experimental

### 2.1. Materials

FAL (99.8%), FA (99.8%), Al_2_O_3_ and Cu(NO_3_)_2_·3H_2_O were purchased from McLean Chemical Reagent Co., Ltd. (Shanghai, China). NH_3_·H_2_O (25 wt%) was purchased from Aladdin Reagent Shanghai Co., Ltd. (Shanghai, China). Isopropanol was purchased from Fengchuan Chemical Reagent Technology Co., Ltd. (Tianjin, China). The deionized water was self-prepared. All reagents were used without further purification.

### 2.2. Catalyst Preparation

The Cu/Al_2_O_3_ catalyst precursor was prepared by the ammonia evaporation method [42]. Firstly, 2.4030 g of Cu(NO_3_)_2_·3H_2_O was dissolved in 140 mL of deionized water. After stirring for 0.5 h, 25 wt% ammonia solution was slowly added to the above solution under stirring to adjust the pH to 12. Then, Al_2_O_3_ (12 g) was added to the above solution, and the suspension was stirred for another 4 h. Thereafter, the mixture was heated to 90 °C to evaporate ammonia until the pH decreased to 7. Finally, the resultant solid was filtered, washed thoroughly with the deionized water and dried at 120 °C for 12 h to obtain the Cu/Al_2_O_3_ catalyst precursor.

The Cu/Al_2_O_3_ catalyst precursor was calcined at 450 °C for 4 h in the different atmospheres, including N_2_ and air in the tube furnace. Before each test, Cu/Al_2_O_3_ catalyst was reduced in 10% vol. H_2_/Ar at 350 °C for 4 h. The calcined and reduced catalysts were denoted as Cu/Al_2_O_3_-N_2_, Cu/Al_2_O_3_-air, Cu/Al_2_O_3_-N_2_-R and Cu/Al_2_O_3_-air-R, respectively.

### 2.3. Catalyst Characterization

In situ X-ray diffraction (XRD) measurement was carried out on a SmartLab SE diffractometer (Rigaku Corporation), and the reaction cell was controlled by PTC-EVO. The patterns were recorded with a 2 Theta range of 20–80° and scan rate of 10 °/min. Transmission electron microscopy (TEM) and high-resolution TEM (HRTEM) were taken on a Tecnai G2 S-Twin F20 TEM microscope (FEI Company) at 200 kV. The textural properties were measured on an ASAP 2460 sorptometer (Micromeritics) at −196 °C. The sample was degassed at 150 °C for 3 h before each test. The specific surface area (S_BET_) was calculated by the Brunauer–Emmett–Teller (BET) method, and the pore volume, as well as the pore size, was calculated by the Barrett–Joyner–Halenda (BJH) model. The actual Cu loading was determined by an inductively coupled plasma atomic mission spectrometer (ICP-AES) on Shimadzu ICPE-9820. X-ray photoelectron spectra (XPS) and X-ray excited Auger electron spectra (AES) were acquired on a Thermo VG Scientific ESCALAB 250 (UK) equipped with Al Kα X-ray excitation. All binding energies were referenced to C 1s (284.8 eV).

H_2_ temperature-programmed reduction (H_2_-TPR) and Cu dispersion measurement were performed on AutoChem Ⅱ 2920 chemisorption analyzer (Micromeritics) equipped with an online thermal conductivity detector (TCD). For H_2_-TPR, the catalyst (~100 mg) was loaded into the U-shaped quartz tube, and pretreatment occurred under Ar flow at 120 °C for 60 min. The signal of H_2_ consumption was subsequently monitored under 10 vol.% H_2_/Ar (50 mL/min) at a heating rate of 10 °C/min from room temperature to 800 °C. Cu dispersion was measured by the technique of N_2_O titration [43,44]. Firstly, the fresh catalyst (~100 mg) was pretreated at 120 °C for 60 min under Ar flow and cooled to 50 °C. Afterward, the sample was heated to 350 °C under 10 vol.% H_2_/Ar gas (50 mL/min) with a rate of 10 °C/min. The area of H_2_ consumption was recorded as X. Then, the tube was cooled down to 50 °C and re-oxidized by 10 vol.% N_2_O/He for 1 h. Finally, the sample was heated to 350 °C under 10 vol.% H_2_/Ar (50 mL/min) with a rate of 10 °C/min. The area of H_2_ consumption was recorded as Y. Cu dispersion and the surface area of surface Cu species per gram catalyst (S(m^2^∙g_cat_^−1^)) were calculated according to the following equations:

(1) Cu dispersion=[2Y / X] ×100%

(2) $$S=(2\;\times \;N_{A}\;\times \;Y)\;/\;(X\;\times \;1.4\;\times \;10^{19}{\times \;M}_{\mathrm{Cu}}\;\times {\;\mathrm{Wt}}_{\mathrm{Cu}}\%)=(1353\;\times \;Y)\;/\;(X\;{\times \;\mathrm{Wt}}_{\mathrm{Cu}}\%)$$

where, M_Cu_, Wt_Cu_% and N_A_ are the molecular weight of Cu, actual Cu loading and Avogadro’s constant, respectively. In addition, 1.4 × 10^19^ is the number of copper atoms per square meter.

In situ diffuse reflectance infrared Fourier transform (DRIFT) spectra of FAL adsorption and hydrogenation were conducted on INVENIO S FTIR (Bruker) equipped with a diffuse reflectance cell and mercury-cadmium telluride (MCT) detector. Before each test, the sample was reduced under 10 vol.% H_2_/Ar at 350 °C for 1 h. After cooling down to 30 °C under N_2_, the background signal was collected. Then, FAL was introduced into the cell with a bubbler using N_2_ as the carrier. Thereafter, the temperature was heated to 120 °C to desorb the physically adsorbed FAL. When the desorption was completed, 10 vol.% H_2_/Ar was introduced for in situ hydrogenation reaction, and the spectra were finally collected every 10 min.

### 2.4. Catalyst Evaluation

The activity of the catalysts was evaluated in a 100 mL stainless-steel batch reactor, which was equipped with mechanical stirrer and temperature controller. Typically, catalyst (0.1 g), FAL (0.5 g) and solvent (30 mL) were added into the reactor. After sealing, H_2_ was charged to replace the internal air for 5 times. Then, the reactor was filled with the desired H_2_ pressure and heated to the predetermined temperature under the continuous mechanical stirring (800 rpm). After the required time, the reactor was naturally cooled down to room temperature, and the mixture was separated by filtration and collected. The products were analyzed by a gas chromatographer (GC9600) equipped with flame ionization detector (FID), and the carrier gas was ultra-high-purity N_2_. The standard solutions of FAL and FA with different concentrations were precisely prepared. External standard method was used to quantify FAL conversion and product selectivity as follows [45,46]:

(3) $$\mathrm{Conversion}\;\left(\%\right)=\frac{\mathrm{Mole}\;\mathrm{of}\;\mathrm{FAL}\;\mathrm{reacted}}{\mathrm{Mole}\;\mathrm{of}\;\mathrm{initial}\;\mathrm{FAL}}\;\times \;100\%$$

(4) $$\mathrm{Selectivity}\;(\%)=\frac{\mathrm{Mole}\;\mathrm{of}\;\mathrm{FA}\;}{\mathrm{Mole}\;\mathrm{of}\;\mathrm{FAL}\;\mathrm{converted}}\;\times \;100\%$$

(5) Yield (%)= Conversion × selectivity

## 3. Results and Discussion

### 3.1. Structure and Morphology

**Figure 2:**
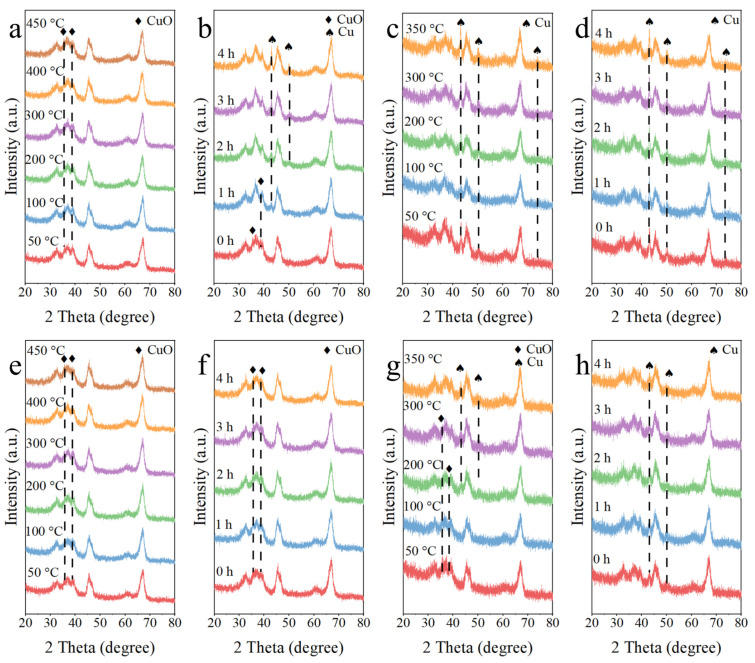
In situ XRD patterns of the Cu/Al_2_O_3_ precursor calcinated in N_2_ (**a**,**b**) and further reduced in H_2_/Ar (**c**,**d**): (**a**) 50–450 °C with different temperatures, (**b**) 450 °C with different time, (**c**) 50–350 °C with different temperatures, (**d**) 350 °C with different time; In situ XRD patterns of the Cu/Al_2_O_3_ precursor calcined in air (**e**,**f**) and further reduced in H_2_/Ar (**g**,**h**): (**e**) 50–450 °C with different temperatures, (**f**) 450 °C with different time, (**g**) 50–350 °C with different temperatures, (**h**) 350 °C with different time.

Figure 2a–f show the in situ XRD patterns of the Cu/Al_2_O_3_ catalyst precursor during calcination in N_2_ and air and the following reduction in H_2_/Ar. As shown in Figure 2a, with the increased temperature from 50 to 450 °C in the N_2_ atmosphere, only the CuO species with the characteristic diffraction peaks at 35.6° and 38.7° were observed [43,47]. Here, the CuO species might be derived from the thermal decomposition of Cu(OH)_2_ and [Cu(NH_3_)_x_]^2+^ in the precursor. Figure 2b displays the further thermal treatment in N_2_ atmosphere at 450 °C. The peaks at 43.3° and 50.5° corresponded to the (111) and (200) crystalline planes of Cu^0^, respectively [48]. Notably, the intensity of the diffraction peaks ascribed to the CuO species decreased gradually with the extended time (after 2 h, the peaks disappear), while the diffraction peaks indexed to the Cu^0^ species appeared after 1 h and became sharper with the prolonging time. Figure 2c displays the reduction process of the Cu/Al_2_O_3_-N_2_ catalyst in H_2_/Ar atmosphere with the increased temperature from 50 to 350 °C. Observably, the intensity of the diffraction peaks assigned to Cu^0^ gradually enhanced with increasing the reduction temperature, implying a further reduction of the CuO species. Figure 2d shows the patterns of the Cu/Al_2_O_3_-N_2_ catalyst reduced for another 4 h at 350 °C. It was noted that the diffraction peaks of Cu^0^ were almost unchanged, suggesting the high anti-sintering ability of the Cu/Al_2_O_3_ catalyst [49].

From in situ XRD patterns in Figure 2e,f during the calcination in air, one can note that only the diffraction peaks of CuO species were present, and no diffraction peaks of Cu^0^ appeared. When the Cu/Al_2_O_3_-air catalyst was reduced by H_2_/Ar (Figure 2g), the intensity of the diffraction peaks attributed to CuO decreased with increasing the reduction temperature, and the diffraction peaks of Cu^0^ species came to exist at 200 °C. When the reduction temperature was increased to 300 °C, the diffraction peaks of CuO completely disappeared. The intensity of the diffraction peaks corresponding to Cu^0^ continuously increased when the reduction temperature rose to 350 °C. As shown in Figure 2h, the diffraction peaks intensity of Cu^0^ remained stable after 3 h reduction at 350 °C. The aforementioned analysis indicates that the presence of Cu^0^ species during the calcination in N_2_ may have been to the generation of Cu(NH_3_)_4_(NO_3_)_2_ in the catalyst precursor prepared by ammonia evaporation method. The thermal decomposition of Cu(NH_3_)_4_(NO_3_)_2_ at a high temperature can produce reduction by NH_3_, which could reduce CuO to Cu^0^. Therefore, the Cu/Al_2_O_3_ precursor was easier to reduce to the lower valence state in the N_2_ atmosphere.

**Figure 3:**
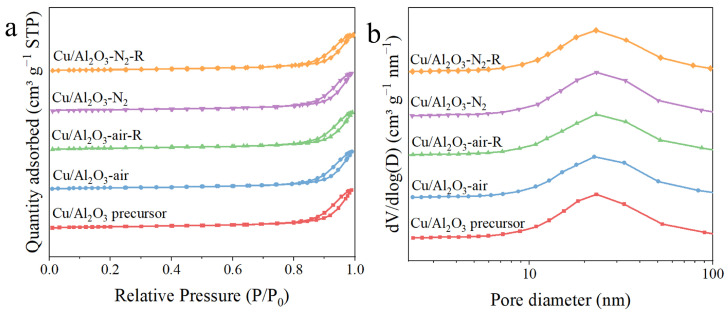
(**a**) N_2_ adsorption-desorption isotherms and (**b**) pore size distributions of the Cu/Al_2_O_3_ precursor and catalysts.

To study the textural properties of the Cu/Al_2_O_3_ precursor and the employed catalysts, N_2_ physical adsorption-desorption experiments were carried out (Table 1 and Figure 3). According to the IUPAC classification [50], all the isotherms exhibited type IV with H3 hysteresis loop (Figure 3a), indicating the typical mesoporous structure [48,51]. As shown in Figure 3b and Table 1, the specific surface areas, pore volumes and average pore sizes of the precursor and the catalysts, including the calcined and reduced samples, were very similar. It illustrates that the calcination atmosphere and further reduction did not affect the textural properties of the Cu/Al_2_O_3_ catalyst [52].

**Figure 4:**
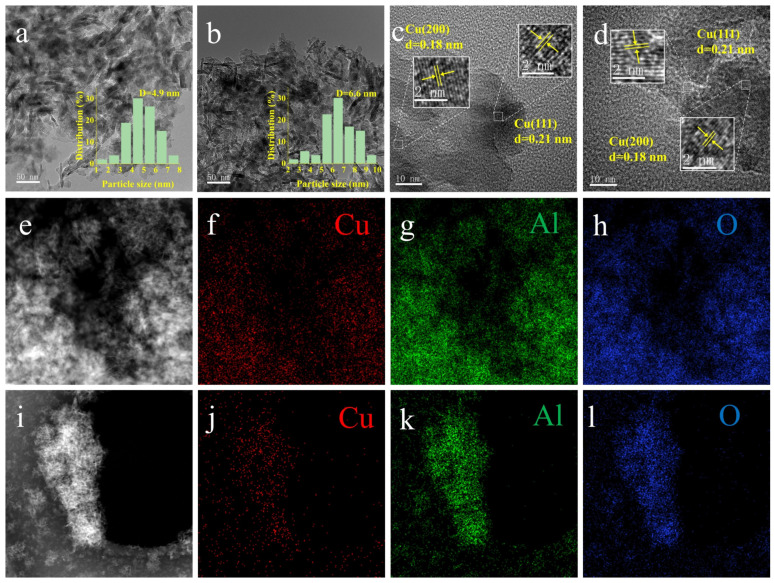
TEM images: (**a**) Cu/Al_2_O_3_-N_2_-R, (**b**) Cu/Al_2_O_3_-air-R. HRTEM images: (**c**) Cu/Al_2_O_3_-N_2_-R, (**d**) Cu/Al_2_O_3_-air-R. HAADF-STEM images: (**e**) Cu/Al_2_O_3_-N_2_-R, (**i**) Cu/Al_2_O_3_-air-R. EDS mapping images: (**f**–**h**) Cu/Al_2_O_3_-N_2_-R, (**j**–**l**) Cu/Al_2_O_3_-air-R.

TEM and HRTEM were employed to investigate the size of Cu nanoparticles and the morphology of the reduced Cu/Al_2_O_3_ catalysts. As shown in Figure 4a,b, the average size of Cu nanoparticles was 4.9 and 6.6 nm for Cu/Al_2_O_3_-N_2_-R and Cu/Al_2_O_3_-air-R, respectively. That is, the average particle size of the Cu species on Cu/Al_2_O_3_-N_2_-R was smaller and more uniformly dispersed than that on Cu/Al_2_O_3_-air-R. The lattice fringe spacing was measured to be 0.21 and 0.18 nm in the HRTEM images (Figure 4c,d), corresponding to the (111) and (200) crystalline plane of Cu, respectively [48]. The assignment of the above crystalline planes is consistent with the XRD results. Moreover, the high-angle annular darkfield scanning transmission electron microscopy (HAADF-STEM) and the corresponding elemental mapping images (Figure 4f–h,j–l) confirm that Cu, O and Al were homogeneously distributed on the Cu/Al_2_O_3_ catalysts [53].

The actual Cu loadings on the catalysts determined by ICP-AES are listed in Table 1. The actual loading of 4.3 wt.% was lower than the theoretical loading of 5 wt.%, which may have been caused by the incomplete precipitation and washing during the preparation [42]. The actual loadings on the different catalysts were the same, which suggests that the calcination atmosphere and reduction treatment could not have resulted in the metal loss and thus did not affect the experimental results and conclusions. Moreover, the dispersion (D_Cu_) and specific surface area of Cu (S_Cu_) on the catalysts were calculated (Table 1). The results show that the dispersion on Cu/Al_2_O_3_-N_2_-R catalyst was 48.3%, which was much higher than that on Cu/Al_2_O_3_-air-R catalyst, and the specific surface area of Cu was 14.1 m^2^_Cu_/g_cat_. The Cu dispersion and specific surface area on Cu/Al_2_O_3_-air-R catalyst were only 21.5% and 6.3 m^2^_Cu_/g_cat_, respectively. The data are consistent with the TEM results. In conclusion, the catalyst calcined in the N_2_ atmosphere was more favorable for the dispersion and reduction of Cu species.

### 3.2. Surface Chemical State

**Figure 5:**
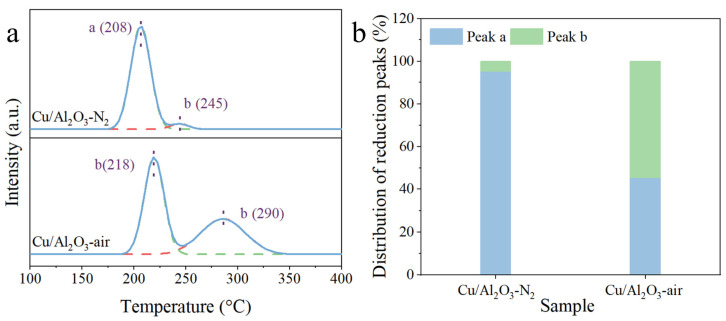
(**a**) H_2_-TPR profiles and (**b**) reduction peaks distribution of the Cu/Al_2_O_3_-N_2_ and Cu/Al_2_O_3_-air catalyst.

H_2_-TPR was performed to study the reduction behavior of the calcined Cu/Al_2_O_3_ catalysts, and the profiles are given in Figure 5a. Apparently, the calcination atmosphere significantly affected the reduction behaviors of the CuO species. The reduction peaks centered at 208 °C/218 °C (a) and 245 °C/290 °C (b) were observed for the Cu/Al_2_O_3_ catalysts [28]. The peak a was attributed to the reduction of the highly dispersed CuO species, and the peak b was related to the reduction of the small-sized CuO species [54,55]. The temperature of the peak a for Cu/Al_2_O_3_-N_2_ was lower than that for Cu/Al_2_O_3_-air. This indicates that the former was more easily reduced, which may have been due to its higher Cu dispersion. Peak fitting and integration were performed to better quantify the percentage of different CuO species, and the quantitative results are plotted in Figure 5b. Cu/Al_2_O_3_-N_2_ had a higher proportion of highly dispersed CuO, accounting for 95.1% of all Cu species, while Cu/Al_2_O_3_-air only contained 45.4% of highly dispersed CuO. This again verifies that for the Cu/Al_2_O_3_ precursor, the calcination in N_2_ was more favorable for the dispersion of Cu species, which are easier to reduce. The analysis is in good agreement with the TEM and N_2_O titration results.

**Figure 6:**
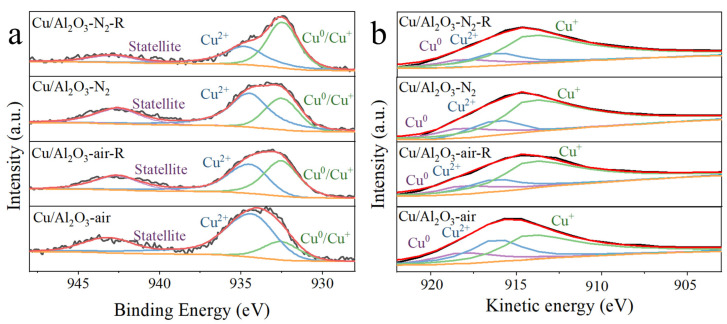
Distribution of Cu species over the employed Cu/Al_2_O_3_ catalysts: (**a**) Cu 2p_3/2_ XPS spectra; (**b**) Cu AES spectra.

To further examine the chemical state of Cu species, XPS was also conducted, and the spectra are displayed in Figure 6a. The spectra at the region of 930.0–937.9 eV were fitted to two characteristic peaks, where the feature at 932.5 eV corresponded to the low oxidation state species (Cu^+^/Cu^0^), and the one at 934.4 eV was assigned to the Cu^2+^ species [54]. According to the fitting results (Table 2), the ratio of (Cu^+^/Cu^0^)/Cu on the surface of Cu/Al_2_O_3_-air was only 21.8%, but the Cu/Al_2_O_3_-N_2_ reached 37.1%. After reduction in H_2_/Ar, the ratio of (Cu^+^/Cu^0^)/Cu increased to 48.8% and 60.8%, respectively. This suggests that the Cu/Al_2_O_3_ catalyst calcined in the N_2_ atmosphere was more easily reduced to the low oxidation state, which is consistent with the H_2_-TPR results.

It is difficult to make a clear distinction between Cu^+^ and Cu^0^ on the basis of XPS analysis [56]. Thus, the Cu AES spectra were also recorded (Figure 6b). Three peaks were extracted by peak fitting in the Kinetic energy (KE) range of 903-922 eV, including the characteristic of Cu^0^ (KE = 918.3 eV), Cu^2+^ (KE = 916.3 eV) and Cu^+^ (KE = 914.3 eV) species [57]. Further quantitative analysis of the fitted peaks is listed in Table 2. The Cu^+^ and Cu^0^ ratio of Cu/Al_2_O_3_ catalyst calcined in air atmosphere were 13.8% and 8.0%, respectively. However, the Cu^+^ and Cu^0^ content of Cu/Al_2_O_3_-N_2_ reached higher levels of 27.4% and 9.7%. It further indicates that the calcination in the N_2_ atmosphere was more favorable for the generation of low valence Cu species, which is in accordance with the results of in situ XRD and TPR. After reduction by H_2_/Ar atmosphere, the Cu^+^ and Cu^0^ content on both Cu/Al_2_O_3_-air-R and Cu/Al_2_O_3_-N_2_-R further increased, where on the former, an increase to 38.6% and 10.2% was observed, respectively, and on the latter, the Cu^+^ and Cu^0^ ratio had the highest values of 45.3% and 15.5%, respectively. According to previous reports, high Cu^+^ and Cu^0^ ratio facilitates the selective hydrogenation of FAL [24,48,54].

Full-spectrum analysis of XPS spectra was performed. The surface Cu/Al atomic ratios of Cu/Al_2_O_3_-N_2_-R and Cu/Al_2_O_3_-air-R were 0.056 and 0.092, respectively. The surface Cu/Al atomic ratio of Cu/Al_2_O_3_-N_2_-R was lower, indicating that Cu nanoparticles were highly dispersed on the Al_2_O_3_ support. The small Cu nanoparticles easily entered the support pores, leading to a decrease in the surface Cu/Al atomic ratio. However, the Cu/Al_2_O_3_-air-R dispersion was poor. A large number of Cu nanoparticles aggregated on the support surface, resulting in a high surface Cu/Al atomic ratio. The above results are in good agreement with the N_2_O titration and TEM results, which prove that Cu/Al_2_O_3_-N_2_-R had higher dispersion.

### 3.3. Catalytic Performance

The selective hydrogenation of FAL over the employed Cu/Al_2_O_3_ catalysts was examined in the stainless-steel batch reactor, and the obtained data are given in Table 3. FA yield over the Cu/Al_2_O_3_ catalysts showed the following trend: Cu/Al_2_O_3_-N_2_-R > Cu/Al_2_O_3_-N_2_ > Cu/Al_2_O_3_-air-R > Cu/Al_2_O_3_-air. Cu/Al_2_O_3_ precursor calcined in air (Cu/Al_2_O_3_-air) had very low FAL conversion and FA selectivity. Even after reduction (Cu/Al_2_O_3_-air-R), the improvement of the catalytic activity was still very limited, where FAL conversion and FA selectivity were only 26.7% and 85.8%, respectively. Interestingly, the precursor calcined in N_2_ had the relatively superior activity. FAL conversion reached 42.7%, and FA selectivity was even 99.9% over Cu/Al_2_O_3_-N_2_. After reduction, the activity was greatly enhanced. Specifically, FAL conversion and FA selectivity reached 99.9% over Cu/Al_2_O_3_-N_2_-R.

The evaluation data indicate that the calcination atmosphere significantly affected the performance of Cu/Al_2_O_3_ catalyst for FAL hydrogenation. Compared with the Cu/Al_2_O_3_-air-R, the FAL conversion and FA selectivity of Cu/Al_2_O_3_-N_2_-R were greatly improved. In general, among the Cu^0^ and Cu^+^ active hydrogenation species on the catalyst surface, the Cu^0^ species contributed the dissociation and activation of H_2_ [27]. The above in situ XRD, TEM, N_2_O titration and H_2_-TPR characterization results demonstrate that Cu/Al_2_O_3_ catalyst calcined in the N_2_ atmosphere was more favorable for the dispersion and reduction of Cu species. This facilitated the reduction of CuO to the lowest valence state of the Cu^0^ species. The XPS results were the same as the above conclusion. The Cu^0^ content over Cu/Al_2_O_3_-N_2_-R was the highest, accounting for 15.5%. Then, Cu^+^ species could act as Lewis acid sites and polarize the carbonyl group (C=O), thus promoting the formation of FA [24]. The Cu AES spectra fitting results show that Cu/Al_2_O_3_-N_2_-R also contained the highest content of Cu^+^ species, accounting for 45.3%. Compared to Cu/Al_2_O_3_-air, the Cu/Al_2_O_3_ catalyst calcined in the N_2_ atmosphere had higher Cu dispersion and higher content of Cu^+^/Cu^0^, resulting in the improved FA conversion and FAL selectivity. After reduction, Cu/Al_2_O_3_-N_2_-R obtained the highest content of Cu^+^ and Cu^0^ active hydrogenated species, and the FAL conversion was further improved. Cu^0^ and Cu^+^ synergistically catalyzed the selective hydrogenation of FAL to prepare FA. Therefore, Cu/Al_2_O_3_-N_2_-R had the highest catalytic activity.

**Figure 7:**
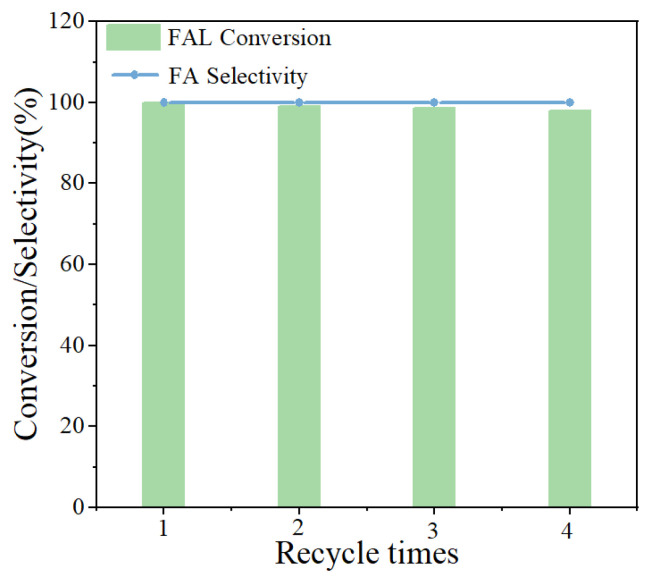
Reusability performance of Cu/Al_2_O_3_-N_2_-R. Reaction conditions: Cu/Al_2_O_3_-N_2_-R (0.1 g), FAL (0.5 g), isopropanol (30 mL), 120 °C, H_2_ pressure (1 MPa), time (2 h).

The reusability of the Cu/Al_2_O_3_-N_2_-R catalyst for FAL hydrogenation was also tested. After every cycle, the catalyst was washed with isopropanol and water to remove the adsorbed organics on the surface and dried overnight in a vacuum oven at 60 °C. As displayed in Figure 7, FAL conversion and FA selectivity was not obviously declined after four recycles. These results imply that the catalyst possessed a good reusability performance.

Table 4 gives a brief comparison of the performance over the representative Cu-based catalysts for FAL hydrogenation to FA using the batch reactor. Notably, the present work provides an efficient FAL hydrogenation catalyst under the relatively mild conditions. Therefore, the strategy in this work has certain advantages for the selective hydrogenation of FAL catalyzed by non-noble metal catalysts.

### 3.4. Reaction Mechanisms

**Figure 8:**
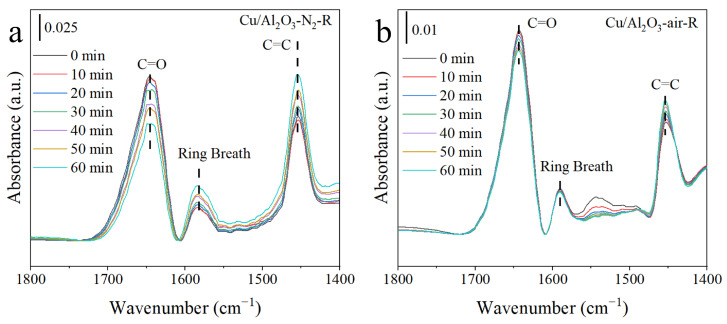
In situ DRIFT spectra of FAL adsorption and hydrogenation at 120 °C.

Figure 8a shows the in situ DRIFT spectra of FAL adsorption at 120 °C. The observed bands located at 1643 cm^−1^ were attributed to the C=O stretching vibration [71], and the bands at 1592 cm^−1^ and 1452 cm^−1^ corresponded to the furan ring breath and C=C characteristic vibrations, respectively [51,71]. Compared with the v(C=O) for gaseous FAL, the significant red shift in v(C=O) from 1670 cm^−1^ to 1643 cm^−1^ implies that FAL was linearly chemical adsorbed in the η^1^(O) configuration on the catalysts [52]. This facilitates FAL hydrogenation towards the production of FA. Cu/Al_2_O_3_-N_2_-R had larger C=O peak intensity, which indicates that Cu/Al_2_O_3_-N_2_-R was more favorable for C=O chemical adsorption. Moreover, in situ DRIFT spectra of FAL hydrogenation are also provided in Figure 8b to further understand the evolution of C=O hydrogenation. Apparently, the strong characteristic bands assigned to C=O declined faster on Cu/Al_2_O_3_-N_2_-R with the continuous inflow of H_2_ than the Cu/Al_2_O_3_-air-R catalyst, indicating the higher catalytic activity of Cu/Al_2_O_3_-N_2_-R. This is consistent with experimental data on FAL hydrogenation.

## 4. Conclusions

In summary, the Cu/Al_2_O_3_ catalysts pretreated by calcining in the N_2_ and air atmosphere were prepared and applied for FAL liquid-phase hydrogenation. The activity of the catalyst calcined in air was lower than that calcined in N_2_, which was related to the generated lower valence state during the calcination process on the latter. After reduction, the performance of FAL selective hydrogenation to FA was proliferated. Specially, the Cu/Al_2_O_3_-N_2_-R catalyst exhibited the most excellent performance. At 120 °C, 1 MPa H_2_, 2 h, both FAL conversion and FA selectivity over Cu/Al_2_O_3_-N_2_-R reached 99.9%, which was comparable to the reported Cu-based catalysts. The comprehensive characterization results based on in situ XRD, TEM, N_2_O titration, H_2_-TPR and XPS demonstrate that the Cu/Al_2_O_3_ catalyst calcined in the N_2_ atmosphere was more favorable for the dispersion and reduction of Cu species and the reduction process could produce more Cu^+^ and Cu^0^ species. Thus, Cu/Al_2_O_3_-N_2_-R had the highest content of Cu^+^/Cu^0^ active hydrogenation species among the employed catalysts, which provides a reasonable explanation for its efficient activity under the relatively mild conditions. This work designs a simple strategy for optimizing the reactivity of Cu-based catalysts and is conducive to efficiently utilizing biomass and its derivatives to develop a renewable energy system.

## Figures and Tables

**Table 1:** Physicochemical properties of the Cu/Al_2_O_3_ precursor and the employed catalysts.

Sample	S_BET_ (m^2^/g) ^a^	V_pore_ (cm^3^/g) ^a^	D_pore_ (nm) ^a^	Cu Loading (wt.%) ^b^	D_Cu_ (%) ^c^	S_Cu_ (m^2^_Cu_/g_cat_) ^c^
Cu/Al_2_O_3_ precursor	131.5	0.80	20.5	-	-	-
Cu/Al_2_O_3_-N_2_	125.1	0.80	20.7	4.3	-	-
Cu/Al_2_O_3_-N_2_-R	128.5	0.76	19.9	4.3	48.3	14.1
Cu/Al_2_O_3_-air	125.0	0.78	21.1	4.3	-	-
Cu/Al_2_O_3_-air-R	127.2	0.77	20.8	4.3	21.5	6.3

^a^: Measured by N_2_ adsorption; ^b^: Determined by ICP-AES; ^c^: Calculated by N_2_O titration.

**Table 2:** Surface element compositions on the Cu/Al_2_O_3_ catalysts.

Catalyst	(Cu^0^ + Cu^+^)/Cu(%) ^a^	Cu^+^/Cu(%) ^b^	Cu^0^/Cu(%) ^b^
Cu/Al_2_O_3_-N_2_	37.1	27.4	9.7
Cu/Al_2_O_3_-N_2_-R	60.8	45.3	15.5
Cu/Al_2_O_3_-air	21.8	13.8	8.0
Cu/Al_2_O_3_-air-R	48.8	38.6	10.2

^a^: calculated from the XPS data. ^b^: calculated from the AES data.

**Table 3:** Hydrogenation performance of FAL to FA over the employed Cu/Al_2_O_3_ catalysts.

Entry	Catalyst	FAL Conversion (%)	FA Selectivity (%)	FA Yield (%)
1	Cu/Al_2_O_3_-N_2_	42.7	99.9	42.7
2	Cu/Al_2_O_3_-N_2_-R	99.9	99.9	99.9
3	Cu/Al_2_O_3_-air	14.3	62.2	8.9
4	Cu/Al_2_O_3_-air-R	26.7	85.8	22.9

Reaction conditions: (0.1 g), FAL (0.5 g), isopropanol (30 mL), temperature (120 °C), time (2 h), H_2_ pressure (1 MPa).

**Table 4:** A brief comparison of the performance over the representative Cu-based catalysts for FAL hydrogenation to FA using the batch reactor.

Catalyst	Reaction Conditions	Con. (%)	Sel. (%)	Yield (%)	Ref.
Cu_2_Ni_1_AlO_y_	120 °C, 1.6 MPa H_2_, 1.5 h	98.0	99.0	97.0	[58]
10Cu-MCM-41-GLY	150 °C, 2 MPa H_2_, 2 h	98.1	80.0	78.0	[59]
Cu/SiO_2_	110 °C, 1 MPa H_2_, 4 h	66.0	100.0	66.0	[60]
Cu/C-400	170 °C, 2 MPa H_2_, 3 h	99.6	99.3	98.9	[61]
Cu/MgO	180 °C, 0.9 MPa	95.0	94.0	89.3	[62]
CuAlO_x_	220 °C, 3.5 MPa H_2_, 5 h	91.0	98.4	89.5	[63]
Na-Cu@TS-1	110 °C, 1 MPa H_2_, 2 h	93.0	98.1	91.2	[64]
Cu/Co_3_O_4_	170 °C, 2 MPa H_2_, 1 h	100.0	67.0	67.0	[65]
NiCu_0.33_/C	120 °C, 1.5 MPa H_2_, 12 h	96.7	93.8	89.6	[66]
7NiCu/ZrO_2_	200 °C, 1.5 MPa H_2_, 4 h	99.5	93.0	93.0	[67]
Ni-Cu	110 °C, 6 MPa H_2_, 2.3 h	50.0	100.0	50.0	[68]
Cu/γ-Al_2_O_3_	130 °C, 4 MPa H_2_, 4 h	64.2	72.3	46.4	[69]
Cu/CeO_2_	210 °C, 0.1 MPa H_2_	83	67	55.6	[70]
Cu/Al_2_O_3_-N_2_-R	120 °C, 1 MPa H_2_, 2 h	99.9	99.9	99.9	This work

## Data Availability

Data will be made available on request.
